# Multidimensional Assessment of Recovery After Total Knee Arthroplasty in Clinical Practice: Critical Narrative Review

**DOI:** 10.2196/84011

**Published:** 2026-02-25

**Authors:** Abderrahmane Boukabache, Nimalan Maruthainar, Vikrant Manhas, Darren Player

**Affiliations:** 1 Division of Surgery and Interventional Science, Faculty of Medical Sciences, University College London, Bloomsbury Campus: 2nd Floor, Charles Bell House, 43-45 Foley Street, London, W1W 7TY, United Kingdom, 44 07946598502; 2 Department of Trauma and Orthopaedics, Royal Free Hospital, London, United Kingdom; 3 Department of Orthopaedics, All India Institute of Medical Sciences, New Delhi, India

**Keywords:** arthroplasty, osteoarthritis, function, outcome, total knee replacement

## Abstract

**Background:**

Total knee arthroplasty (TKA) is the primary treatment for advanced knee osteoarthritis. Despite its clinical success and favorable patient-reported outcome measures (PROMs), approximately 20% to 30% of patients continue to experience persistent functional limitations and muscle weakness. This highlights the need for a comprehensive evaluation of recovery parameters beyond pain and range of motion. Given the wide range of methods available for assessing TKA outcomes, clinicians often select tools based on personal preference and understanding, which may affect accuracy and consistency; for example, the Knee Injury and Osteoarthritis Outcome Score may overestimate function compared to gait analysis studies.

**Objective:**

The aim of this study was to conduct a narrative review focusing on the use, strengths, and limitations of different outcome measures used in routine orthopedic practice to optimize post-TKA evaluation.

**Methods:**

A literature search was conducted in February 2025 across 2 databases (PubMed and Web of Science). Eligible studies included original research articles, systematic reviews, and meta-analyses that focused on validated measures used to evaluate TKA. Case reports, conference abstracts, and studies focused exclusively on surgical techniques were excluded. Themes were identified across studies to structure the results according to types of assessments and clinical applicability.

**Results:**

A total of 6831 studies were retrieved and screened in this review, with 4 themes emerging around muscle mass, strength, performance, and PROMs. The Oxford Knee Score is favored for its ease of use and minimal ceiling effects. Broader tools like the Knee Injury and Osteoarthritis Outcome Score and Western Ontario and McMaster Universities Osteoarthritis Index provide detailed insights but are less practical clinically. For muscle strength, the portable fixed dynamometer showed high reliability and comparability to isokinetic dynamometry. Dual-energy X-ray absorptiometry remains the gold standard for assessing muscle mass, while bioelectrical impedance analysis offers a practical alternative. The 5-Repetition Sit-to-Stand test effectively evaluates lower limb power and speed.

**Conclusions:**

Clinicians should integrate both objective (muscle mass, strength, and performance) and subjective (PROMs) measures to improve TKA recovery assessment. This multidimensional approach has the potential to enhance the accuracy of patient evaluation and supports the development of tailored rehabilitation strategies that address individual deficits and optimize functional outcomes.

## Introduction

Osteoarthritis is a leading cause of pain and disability worldwide, affecting 528 million people as of 2019 [1,2]. Among various treatment options for osteoarthritis, total knee arthroplasty (TKA) stands out for its substantial impact on alleviating chronic knee pain [3]. With advancements in prosthetic design and surgical techniques, TKA has demonstrated high survival rates and relatively low complication risks, making it one of the most successful orthopedic procedures. However, despite these clinical successes, postoperative functional outcomes can vary among patients [4], with approximately 20% to 30% experiencing persistent functional limitations and muscle weakness [5].

Several factors influence recovery following TKA, including surgeon-related aspects such as surgical volume and technique, as well as patient-related variables like preoperative physical conditioning and psychological status. Among these, preoperative quadriceps strength has been identified as a key predictor of postoperative function, directly impacting mobility and the ability to perform daily activities [6]. Nonetheless, frequently used tools to evaluate TKA in orthopedics often rely on measures such as pain assessment and range of motion (ROM) [7], which, while valuable, may not fully capture postoperative recovery or functional capacity.

Evidence suggests that these metrics correlate poorly with objective measures of physical function and do not adequately reflect muscle weakness or biomechanical impairments that persist post-TKA [8]. While patient-related outcome measures (PROMs) provide subjective insights into perceived recovery, they may overestimate functional improvements compared to more objective assessments, such as gait analysis and performance-based tests [9]. This discrepancy underscores the need for a more comprehensive evaluation framework that integrates multiple dimensions of recovery, including muscle mass, muscle strength, and physical performance.

This review aims to critically examine the use, strengths, and limitations of different outcome measures used in routine practice when assessing recovery post-TKA, emphasizing the importance of incorporating objective physical performance metrics alongside PROMs. We hypothesize that a multidimensional approach, combining assessments of muscle mass, strength, physical performance, and PROMs, will provide a more accurate and clinically meaningful evaluation of TKA recovery, ultimately guiding more effective rehabilitation strategies.

## Methods

A literature search of studies focusing on methods assessing TKA outcomes was performed using PubMed and Web of Science databases in February 2025. No limits were applied to publication dates. Inclusion criteria comprised original research, systematic reviews, and meta-analyses. After the removal of duplicates and cross-referencing of articles, we screened titles and abstracts for relevance. Studies were excluded if they were case reports, conference abstracts, or focused exclusively on surgical techniques. Only studies using standardized and validated assessment tools were included to ensure clinical relevance. The methodological quality of included studies was evaluated based on study design, sample size, and clarity of outcome measures.

One author conducted the initial screening of all retrieved abstracts against the inclusion criteria. Full texts of potentially relevant studies were then independently reviewed for eligibility and data extraction. Discrepancies between reviewers were resolved through discussion until consensus was achieved. Extracted data were thematically organized (muscle mass, muscle strength, physical performance, and PROMs), allowing patterns across assessment domains to be identified and synthesized into a structured narrative aligned with the review’s objectives.

Given the narrative nature of this review, our selection was not strictly systematic; instead, we documented the total number of retrieved and screened articles (n=6831).

## Results

### Patient Reported Outcome Measures

There are numerous PROMs available to assess the outcomes of TKA, including: Knee Society Clinical Rating System (KSS), Western Ontario and McMaster Universities Osteoarthritis (WOMAC), Knee Injury and Osteoarthritis Outcome Score (KOOS), and the Oxford Knee Score (OKS) [10-13]. In clinical practice and research, it is essential to prioritize the use of sound PROMs over frequently used ones. The ultimate goal herein is to find a balance between standardized measures and specific contextually relevant PROMs in the field of TKA.

The KSS and OKS PROMs are shorter than the KOOS and WOMAC, with 10 and 12 items, respectively. The OKS uses a 4-week recall period, while the patient-reported component of the KSS is designed to reflect current knee status without a fixed recall window, which may reduce recall bias but limit comparability across time points. The OKS is solely derived from patient input, while the KSS comprises a PROM section completed by the patient and an informational-clinical section for the surgeon. Only the PROM section of the KSS is used to produce a psychometrically valid knee score. This separation sought to allow the functional PROM section to be independently assessed from the clinical section and confounding factors such as age or comorbidities. Notably, Martimbianco et al [14] reported considerable inter- and intraexaminer variability in the clinical-reported section of the KSS, which raises concerns about its reliability and consistency in clinical use. In contrast, the KOOS and WOMAC are larger instruments, with 42 and 33 items, respectively. They assess symptoms, stiffness, pain, and activities of daily living (ADL), but also include additional domains such as sports or recreation and quality of life. Like the OKS, these instruments are totally focused on the patient and their self-reported experiences. The WOMAC uses a 48-hour recall period, which minimizes recall bias and improves measurement precision; however, this narrow timeframe may fail to reflect symptom variability and functional fluctuation over longer recovery periods following TKA. In contrast, the KOOS uses a 1-week recall period, providing a broader representation of knee symptoms and functional limitations. Nevertheless, this longer recall window may increase susceptibility to recall bias and potentially reduce sensitivity to short-term clinical change.

All 4 PROMs assess pain and activity function, but they differ in how they capture pain information. The OKS and KSS both include a single item that captures the patient’s general level of pain. However, the OKS asks further pain information in the context of general activities, such as work interference and walking. This is particularly important to assess as movement is associated with changes in pain, from subtle changes in muscle coordination to complete avoidance of joint function [15]. In contrast, the KOOS and WOMAC both include a section dedicated to assessing pain experienced during specific activities, with nine identical items. This encourages the patient to recall and report on the pain experience during knee maneuvers such as knee twisting and straightening. The KOOS is an extension of the WOMAC, which justifies the similarities in the items. It is used in younger and/or more active patients who typically perform demanding twisting movements in their ADL. By assessing knee pain during movements like twisting and straightening, changes in functional outcomes over time can be tracked. This is especially relevant post-TKA, where pain during specific activities is directly linked to the functionality of the joint and the surrounding muscles [16].

The only resemblance when assessing function across the 4 PROMs is the patient’s comfort level when handling stair ascent and descent. This likely reflects the universal importance of these activities in daily life and the significant impact that knee function has on an individual’s mobility and well-being. However, each PROM also has unique items that assess different aspects of knee function and quality of life.

The OKS, KOOS, and WOMAC all assess the knee concerning various ADL, such as kneeling, transportation, domestic work, and bathroom activities. The OKS and KSS share only the item related to the distance the patient can walk. The KSS and items within the symptoms section of both KOOS and WOMAC ask about the patient’s ability to fully bend and extend the knee, as well as objective measures of flexion contracture and extension lag. The use of similar items and domains across these scores can lead to greater consistency in assessing knee function and symptoms. Furthermore, consistency is important for tracking changes over time and comparing outcomes across different patients and studies. The KOOS is unique in that it assesses ADL related to sports and recreation and includes items that ask about quality of life. While similarities can provide consistency, differences between PROMs may capture important nuances specific to each condition.

The KSS evaluates 4 distinct domains: clinical, functional, satisfaction, and fulfillment of expectation. A notable feature is the inclusion of both high-demand tasks and 3 patient-selected priority activities from a predefined list, aiming to tailor the assessment to individual goals and enhance the relevance of functional evaluation. This patient-led component is designed to offer a more individualized perspective on recovery and may help inform more targeted postoperative rehabilitation. However, despite these strengths, the validity of the KSS has been questioned, particularly in the context of TKA outcome measurement. Several studies have noted limitations, including Ghanem et al [17], who highlighted concerns in revision TKA scenarios, and Vogel et al [18], who found the system less responsive than broader tools like the 36-Item Short Form Survey and WOMAC. One key criticism lies in the item-selection process: patients were not involved in the development of the tool, which may result in content that does not fully reflect patient priorities or lived experiences. Bach et al [19] further noted that the relatively limited item pool may constrain the scale’s ability to capture the full spectrum of functional outcomes. This lack of patient input and limited item scope could contribute to a misalignment between what the tool measures and what matters most to patients, ultimately impacting its content validity and clinical use [17,18,20].

Ghanem et al [17] highlight that 2 very different patients could receive the same clinical score on the KSS. For instance, a patient with a stiff, pain-free, well-aligned knee and a patient with mild pain, excellent knee motion, and normal alignment may both receive a similar score, even though they have very different levels of function in their daily lives. To address these concerns, a revised Knee Society Scoring System (2011 Knee Society Score) was developed and validated as a more reliable measure for assessing outcomes in TKA procedures [21,22]. Albeit, a revised outcome measure, PROMs have inherent limitations in that they rely primarily on self-reported data, which may not always align perfectly with objective measures of physical function. This is where muscle function analysis could come into play. It offers an objective assessment of a patient’s physical capabilities, including muscle strength, range of motion, and muscle activation patterns. By quantifying these aspects, health care providers can validate and complement the patient’s self-reported symptoms and limitations with tangible data. This objective information not only supports the patient’s reported experiences but also provides a more complete picture of their health status.

Despite its validity issues, the KSS is still popular among clinical researchers [23], perhaps for including alignment and ROM measurement. Proper coronal alignment of the components in TKA has been shown in the literature to be critical for implant survival rate. Additionally, knee ROM is a crucial indicator of successful TKA and is necessary for many ADLs [24]. While ROM is commonly measured and reported, the significance of isolated muscle function has been overlooked here. Capin et al [25] found quadriceps strength in particular to be a critical factor in TKA recovery and considered a rate-limiting step. This limited quantification of isolated muscle function is an area that warrants attention and improvement in the care of patients who underwent TKA.

The OKS was developed specifically for evaluating the outcomes of TKA. The simplicity and shortness of the questionnaire make it an attractive option for clinicians, and this may in part have contributed to its broad use in cohort studies and joint replacement registers [26,27]. This intentional oversimplification of the questionnaire highlights a lack of scope and sophistication to adequately capture the complex, interrelated issues experienced by many patients, such as joint stiffness, muscle weakness, and instability. To address these limitations, the use of a combination of assessment tools and methods has been suggested [28]. This could include performance-based functional tests, isolated muscle function tests, muscle mass measurement, and imaging studies.

Another consideration is that OKS has a high response rate when compared to other PROMs [10]. Analysis of the National Health Service PROMs dataset of 72,154 OKS concluded that OKS does not exhibit a ceiling or a floor effect at 6 months [29]. Marx et al [30] reported a ceiling effect of 22% at 12 months following surgery, but this could be attributed to patients achieving an optimal outcome rather than a limitation of the OKS. Nevertheless, this could indicate that the OKS may be more appropriate for short-term outcomes and might inadequately reflect the long-term burden. Although it has been used in randomized controlled trials to assess knee arthroplasty long-term outcomes [31].

The WOMAC, developed in 1982, has undergone multiple revisions since and has been validated for TKA clinical trials [32-34], with ceiling effects at 6 months and 12 months for patients who underwent TKA [35]. The KOOS was an upgrade to the WOMAC to effectively capture the requirements of younger and more active individuals with knee injury or osteoarthritis. Several studies have shown that the KOOS is more sensitive and responsive than the WOMAC in younger or more active patients with knee injury or knee osteoarthritis [36]. Roos and Toksvig-Larsen [35] evaluated the outcome of 105 patients (mean age of 71) after TKA and found 74% of all Sport/Recreation items were considered to be “not applicable.” The floor effects of approximately 48% likely reflect the high-demand activities, such as sports and recreation, which may be more relevant for younger, more active adults undergoing TKA.

A systematic review of the literature performed by Collins et al [37] found, as it was intended, the KOOS’s ADL subscale has better content validity for older patients, and Sport/Recreation has better content validity for younger patients. Increasing ceiling effects from 6 to 12 months, particularly in the pain domain, may suggest that some patients have reached a plateau in their recovery, but also raises questions about the sensitivity of the measurement tool.

According to the Consensus-Based Standards for the Selection of Health Measurement Instruments (COSMIN), which define internationally accepted criteria for evaluating the validity, reliability, and responsiveness of PROMs, the OKS meets COSMIN requirements and is recommended for use as a TKA outcome measure. Pain and function subscales of WOMAC and KOOS also demonstrate adequate measurement properties when evaluated as standalone subscales. However, the KSS does not consistently fulfill all COSMIN criteria and is not classified among the instruments meeting minimum standards for psychometric validation [38]. Several studies have evaluated the performance of different PROMs in measuring outcomes following lower extremity joint replacement surgery. Harris et al [39] identified the OKS and WOMAC as the best-performing PROMs specific to the lower extremity. The study assessed the validity, reproducibility, and acceptability of the scoring systems. Similarly, Alviar et al [40] in their review found OKS and WOMAC to be the best PROMs after assessing the quality of patient-reported outcome scoring systems. Collins and Roos [41] analyzed attributes of the 11 most frequently used PROMs for total hip replacement and TKA and considered KOOS, WOMAC, and OKS to be good PROMs. These PROMs aid in monitoring progress, guiding interventions, and facilitating shared decision-making. Nevertheless, there are limitations, such as the risk of subjective bias and variability in interpretation. Patient responses may be influenced by personal perceptions, mood, cognitive state, or cultural differences. Obtaining consistent and accurate data can be challenging, especially if patients struggle to recall specific details over time. To gain a more thorough understanding of the impact of TKA, it is crucial to integrate objective measures of muscle strength and functional assessments, particularly in addressing muscle weakness.

### Strength Measurements

The indirect assessment of muscle function in any of the PROMs may not provide sufficient indication of dysfunction, necessitating a comprehensive analysis of objective assessment tools in TKA (Table 1)(Multimedia Appendix 1). To this end, this section will focus on the types of muscle strength assessments that are feasible in a TKA clinical setting.

**Table 1.** Comparative analysis of outcome measures used in total knee arthroplasty (TKA), including patient-reported outcomes, muscle strength, muscle mass, and physical performance tools. Comparison criteria include measurement type, reliability, validity, ease of use, clinical relevance, limitations, and best use case.

Comparison	PROMs^a^ (KSS^b^, WOMAC^c^, KOOS^d^, and OKS)^e^	Muscle strength tests (IKD^f^, PFD^g^, and HHD^h^)	Muscle mass tests (CT^i^, MRI^j^, DXA^k^, and BIA^l^)	Physical performance tests (TUG^m^, 6MWT^n^, 5R-STS^o^, and SCT^p^)
Measurement type	Subjective (patient-reported pain, function)	Objective (muscle force output)	Objective (muscle cross-sectional area or mass)	Objective (functional movement performance)
Reliability	High test-retest reliability but can be influenced by patient perception	High reliability but dependent on test consistency and patient effort	High for CT, MRI, and DXA; moderate for BIA (affected by hydration)	High for TUG, 6MWT, and 5R-STS
Validity	Valid for subjective function but may not correlate with actual muscle strength or mobility	IKD, highest validity. PFD, good validity with proper fixation. HHD, lower validity	Highly valid for assessing atrophy or hypertrophy	Strong correlation with mobility and functional independence
Ease of use	Simple, noninvasive, and patient-friendly	Requires equipment; portable dynamometers are easier than isokinetic ones	CT or MRI is expensive; DXA is more accessible; BIA is easiest	Quick, requires minimal equipment (eg, chair and stopwatch)
Clinical relevance	Useful for tracking patient-perceived recovery	Directly assesses quadriceps and hamstring strength, critical for TKA recovery	Helps detect muscle loss post-TKA, which can affect long-term function	Strong predictor of fall risk, mobility, and independence
Limitations	Subjective, may not reflect real functional capacity	Requires patient cooperation; costly for isokinetic dynamometers	Expensive (CT and MRI), radiation exposure (CT and DXA), less precise (BIA)	Can be influenced by patient motivation, fatigue, or comorbidities
Best use case	Tracking patient-perceived progress	Measuring post-TKA quadriceps or hamstring recovery	Evaluating long-term muscle loss and sarcopenia	Assessing mobility and real-world function

a PROM: patient-reported outcome measure.

b KSS: Knee Society Score.

c WOMAC: Western Ontario and McMaster Universities Osteoarthritis Index.

d KOOS: Knee Injury and Osteoarthritis Outcome Score.

e OKS: Oxford Knee Score.

f IKD: isokinetic dynamometer.

g PFD: portable fixed dynamometer.

h HHD: handheld dynamometry.

i CT: computed tomography.

j MRI: magnetic resonance imaging.

k DXA: dual-energy X-ray absorptiometry.

l iBIA: bioelectrical impedance analysis.

m TUG: Time Up and Go.

n 6MWT: 6-Minute Walk Test.

o 5R-STS: 5-Repetition Sit-to-Stand.

p SCT: Stair Climb Test**.**

When evaluating muscle function, the measured outcomes commonly include muscle strength and power [42]. Various methodologies assess these parameters, ranging from manual muscle testing to more complex and expensive isokinetic assessments. However, no general consensus exists regarding the preferred method of assessing muscle function in orthopedic practice, as each approach has distinct advantages and limitations [43].

**Figure 1.**
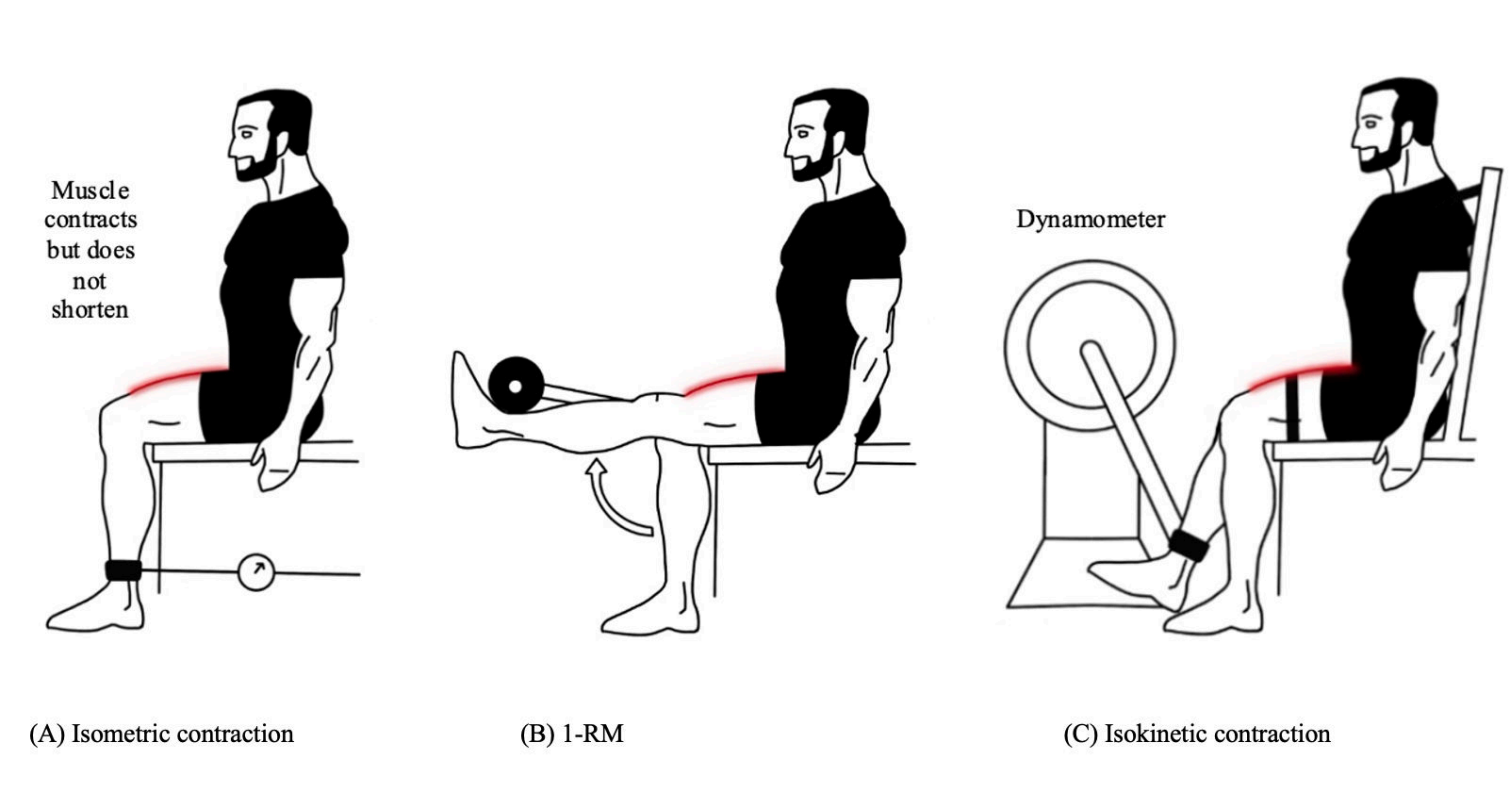
Illustration of 3 common muscle strength assessment methods used in total knee arthroplasty (TKA) recovery: (A) isometric contraction—muscle generates force without joint movement; (B) one-repetition maximum (1-RM)—maximal weight lifted once with proper form; and (C) isokinetic contraction—muscle contracts at a constant speed throughout the range of motion using a dynamometer.

Isometric strength assessment measures the maximum force generated during a contraction in which the muscle length remains constant (Figure 1A). Although isometric contractions are relatively uncommon in daily activities, their clinical relevance lies in their ability to predict functional capacity, particularly in older individuals and those with significant functional impairments [44]. Furthermore, isometric strength assessments have demonstrated high reliability across different orthopedic populations [45,46]. Isometric strength assessments can be easily performed using a dynamometer or force plate, and the individual is instructed to push or pull against an immovable object. Portable fixed dynamometer (PFD) has demonstrated high reliability (intraclass correlation coefficient [ICC] >0.90) and validity in measuring strong muscle contractions such as knee extension [47-49]. Importantly, studies have shown that knee muscle strength measurements using a PFD highly correlate with those obtained from an IKD, making PFD a viable alternative for clinical assessment [50].

Toonstra and Mattacola [51] compared the reliability of IKD, PFD, and handheld dynamometry for isometric knee strength assessment. Their findings revealed high test-retest reliability for both IKD and PFD, while handheld dynamometry showed fair to poor reliability.

The strength of IKD lies in its ability to provide stabilization during testing and standardized protocols, making it the gold standard. However, PFD demonstrated comparable reliability while offering greater portability, ease of use, and cost-effectiveness. This makes PFD an attractive option for routine clinical evaluations, particularly in settings where IKD is not feasible.

Another key advantage of isometric assessments is their safety. Unlike dynamic strength tests, which involve movement and may place stress on healing tissues, isometric assessments minimize joint strain. This makes them particularly suitable for early postoperative assessments, where patient safety is a priority. Given these benefits, isometric strength measurements serve as a practical and reliable method for monitoring recovery following TKA.

One-repetition maximum (1-RM): Muscle strength can also be assessed through the one–repetition maximum (1-RM) test, which determines the maximum load that can be lifted in a single attempt, such as during a squat or leg extension (Figure 1B). This method evaluates muscle strength in a more functional and dynamic context, offering insight into the ability to generate force during real-world movements.

Despite its functional relevance, 1-RM testing has limitations. The requirement for specialized equipment (eg, weights and resistance machines) and the time-intensive nature of testing make it less practical for large patient cohorts. Additionally, 1-RM testing carries a risk of injury, particularly in postoperative populations where patients may not yet be able to safely perform maximal-effort movements. To mitigate this risk, researchers often use submaximal testing protocols to estimate 1-RM values [52].

Isokinetic testing measures muscle strength as the peak torque generated during a contraction performed at a constant angular velocity (Figure 1C). This method provides resistance that matches the individual’s effort throughout the ROM, allowing for maximal force production at different joint angles. Isokinetic assessments are particularly valuable for evaluating muscle imbalances, monitoring rehabilitation progress, and understanding strength deficits following TKA.

Despite these advantages, isokinetic testing has notable limitations. The high cost and lack of portability make it less accessible in many clinical settings [53,54]. Additionally, isokinetic assessments require specialized equipment and trained personnel, making them impractical for routine postoperative evaluations. The potential for joint irritation and discomfort during testing further limits its suitability for early rehabilitation stages.

A key consideration when choosing between isometric and isokinetic testing is clinical feasibility. While isokinetic assessments provide detailed torque-angle relationships, they are not always necessary for functional recovery monitoring. In contrast, isometric testing provides a simple, reliable, and cost-effective means of assessing muscle strength, making it better suited for routine evaluations.

Lauermann et al [55] compared quadriceps strength assessments using isometric, isokinetic, and 1-RM methods in patients who underwent TKA. All 3 methods showed similar validity in detecting strength deficits, suggesting that isometric testing is an effective alternative to more complex assessments. Similarly, Lienhard et al [45] found comparable test-retest reliability across these methods in patients who underwent TKA, reinforcing the viability of isometric assessments in clinical practice.

### Muscle Mass Measurements

Studies have shown a significant correlation between skeletal muscle mass and cross-sectional skeletal muscle area in the extremities with muscle strength or power [56,57]. Hence, assessing these morphological features could be a good way to gauge muscle function. Furthermore, with certain imaging modalities, it may also be possible to determine the extent of sarcopenia, muscle wasting, and the level of fibrosis and fat infiltration—all factors that play a significant role in muscle function.

Computed tomography (CT) is an imaging modality that was introduced over 50 years ago, as the first clinically accepted tool for body composition measurement and served as a gold standard [58]. This method uses X-ray beams to create cross-sectional images of the body, allowing estimation of total body fat, visceral fat, and skeletal muscle mass [59,60]. Despite its early acceptance and high validity for assessing limb muscle cross-sectional area, with excellent test–retest and inter-observer reliability (ICC 0.98‐1.00 for thigh muscle measurements), CT has general limitations such as high costs, the need for skilled technicians, and radiation exposure [61,62]. However, in current practice, CT is rarely used for measuring muscle mass, likely due to its associated limitations and the emergence of alternative, more practical techniques such as magnetic resonance imaging (MRI).

The introduction of MRI in the 1980s replaced the CT scan as the gold standard [63]. Its 3D images of skeletal muscle, fat tissue, and other organs are created by emitting radiofrequency energy from hydrogen atom nuclei in magnetic fields, with signal variations indicating different tissue types [63,64]. This development brought about high-resolution images without radiation exposure, making it suitable for tracking small changes over time, which is beneficial for intervention and observational studies. Advancements in MRI techniques have notably reduced image acquisition times, and modern scanners can accommodate obese individuals. Validated against direct anatomical measurements (*r*=0.97), MRI exhibits high test-retest and inter-observer reliability (2.9%, *r*=0.99% and 2.6%, *r*=0.99) in healthy populations [63,64]. However, limitations in clinical and research settings include high costs, the need for technical expertise, space requirements, infeasibility for patients with claustrophobia or those with MRI-incompatible implanted devices (eg, cardiac pacemakers), and standardization is hampered by the existence of various data acquisition protocols [61,65].

Dual-energy X-ray absorptiometry (DXA) has emerged as a strong alternative to the gold standard for assessing body composition, being relatively cheap compared with CT scan and MRI, and easy to perform. Initially designed for measuring bone mineral density, DXA is now widely used for examining overall body composition and muscle mass [63]. DXA operates by using 2 X-ray beams to differentiate between fat, bone, and lean tissue based on their X-ray absorption properties [66]. This technique has shown a high correlation with both MRI and CT in estimating skeletal muscle mass, indicating its reliability (*r*=0.88 and *r*=0.77‐0.95, respectively) [67-69]. Additionally, DXA test-retest reliability for measures of fat-free mass demonstrated a high correlation (*r*=0.99), with low precision errors ranging from below 1% to 3% [70].

However, DXA does present some limitations. First, it does not directly quantify muscle mass; instead, it calculates lean soft tissue mass or fat-free mass based on the different gray tones observed in the DXA scan. Assumptions are made during this calculation, and factors like dehydration and edema, which can be common in populations with obesity, may interfere with the accuracy of the measurements. Additionally, the lack of standardization across devices, software packages, and versions can yield different results, affecting the reliability and comparability of measurements. Moreover, while DXA involves less radiation exposure compared to CT, there is still some exposure [71,72].

Bioelectrical impedance analysis (BIA) has become widely used for assessing body composition in both clinical and nonclinical settings [73]. BIA operates on the principle that tissues with higher water and electrolyte content, like skeletal muscle, offer less resistance to the passage of a low-voltage electrical current compared to lipid-rich adipose tissue, such as bone. This conductivity difference is exploited by BIA systems to quantify different body compartments. Measurements obtained along with parameters like sex, age, and body weight are then integrated into the population-specific body composition prediction equations [64,72]. The advantages of BIA stem from its noninvasive nature, cost-effectiveness, and ease of use, but limitations include sensitivity to factors like body position, physical exercise, food intake, hydration status, and the need for population-specific equations [74]. A study by Buckinx et al [75] attempted to gauge the reliability of BIA in assessing appendicular lean mass. The results showed high intraoperator reliability, indicated by an ICC of 0.89 when performed by the same operator, and interoperator reliability was also relatively high with an ICC of 0.77 when performed by different operators. However, the study revealed a notable discrepancy when comparing appendicular lean mass assessed by DXA and predicted by BIA, as evidenced by a low ICC of 0.37. Importantly, there exists a potential for a significant prediction error at the individual level with BIA, coupled with a systematic positive bias leading to an overall underestimation of lean body mass measurements [76]. Additionally, hydration status is a frequent confounder in clinical settings, as BIA relies on electrical conductivity that is highly sensitive to fluctuations in body water content, potentially compromising accuracy and limiting its reliability in certain patient populations. Despite these limitations, BIA remains a viable alternative in situations where more precise methods are not feasible.

### Performance Measurements

While measurements of muscle mass and function are important for understanding physiological dysfunction and pathology, assessing physical performance is essential. In this context, functional performance-based tests use an individual’s body weight as resistance, quantifying performance by the time taken or the number of repetitions completed. Several authoritative groups, including The Osteoarthritis Research Society International, Rehabilitative Care Alliance, and the American Physical Therapy Association, recommend performance-based measures for individuals with osteoarthritis and arthroplasty [77-79]. These measures typically include assessments of gait speed tests, Stair Climb Tests (SCT), and Sit-to-Stand (STS) tests, each with distinct yet overlapping focuses.

Gait speed tests primarily assess mobility, endurance, and functional independence by measuring the time required to walk a set distance. They are simple, reliable, and highly predictive of overall recovery, fall risk, and mortality [80]. SCTs focus on functional strength, coordination, and balance, as stair negotiation requires greater knee flexion and quadriceps activation than level walking [81]. SCTs are more sensitive to persistent functional deficits than gait speed tests, as stair climbing is often more challenging postoperatively. STS tests, on the other hand, primarily evaluate lower limb strength and endurance by measuring the ability to transition from sitting to standing multiple times within a set duration or repetition count. STS tests strongly correlate with quadriceps strength and are useful for monitoring functional recovery [82].

While all 3 measures provide valuable insights into TKA recovery, they differ in their primary focus and clinical applicability. Gait speed and SCTs assess mobility in dynamic movement tasks, while STS focuses on lower limb muscle strength in a controlled, stationary task. Gait speed tests are most effective for evaluating overall mobility and endurance, whereas SCTs are ideal for assessing functional strength and dynamic balance [83]. In contrast, STS tests specifically target muscle power and endurance without assessing walking ability or balance [84]. Despite these differences, all three tests share a common goal of evaluating lower limb function and have been shown to be reliable, valid, and responsive to post-TKA functional improvements [78]. However, it is worth noting that both gait speed and SCTs may present logistical challenges in smaller clinical settings due to space constraints and increased time requirements for setup and execution, potentially limiting their routine use in busy outpatient environments.

Dobson et al [78] put forth a set of recommended performance-based measures for individuals diagnosed with hip and knee osteoarthritis or following joint replacement. This selection was based on expert surveys and systematic reviews, considering the evidence supporting the measurement properties of the tests, their feasibility, scoring methods, and expert consensus. The resulting recommended measures included the 30-Second Chair Stand Test (30CST), 40 m Fast-paced Walk Test, SCT, Time Up and Go (TUG), and 6-Minute Walk Test (6MWT). In a subsequent update by Dobson et al [85], the 10 Meter Fast-paced Walk Test and 20-second stair climb test were suggested as alternatives due to complexities in administering the 40-meter Fast-paced Walk and scoring the 11 Step Stair Climb Test.

A task force led by Westby et al [86] created the Total Joint Arthroplasty and Outcome Measures toolkit to be used before and after arthroplasty, which included: 30-second STS, gait speed, stair climb test, single leg stance test, 6MWT, TUG, and functional reach. Prior to the creation of this toolkit, Zeni et al [87] developed the Delaware Osteoarthritis Profile, a comprehensive set of tests that have been effectively used to measure functional performance pre and post knee arthroplasty, which include: TUG, SCT, and 6MWT.

While the literature contains numerous tests, the focus here will be on muscle strength performance tests. STS with its versions has been identified by Bergquist et al [88] in their systematic literature review to be the most appropriate performance-based clinical muscle strength test.

The 30CST is a component of the Senior Fitness Test and involves counting the number of sit-to-stand repetitions completed within a 30-second timeframe. Individuals who are more than halfway through a repetition at the 30-second mark are credited with completing the final repetition. This test has shown good to excellent reliability, with ICCs of approximately 0.84 to 0.92 in clinical populations, and its validity is supported by moderate to strong correlations with leg press strength measures [89].

The 5-Repetition Sit-to-Stand (5R-STS) test has gained widespread use as either a component of the short physical performance battery or as a standalone assessment tool in numerous studies. This test involves performing 5 sit-to-stand repetitions from a standard chair (44‐48 cm height), with timing commencing upon a specific command or the initiation of the first movement and ending when the fifth stand-up is accomplished or when the patient sits down following the fifth stand-up [90]. The reliability of the 5R-STS test has been estimated across 10 studies, yielding a coefficient of 0.81, as reported by Bohannon (2011) [84]. The validity of this test as a measure of lower limb functional muscle strength is supported by its strong correlation with knee extension strength, as demonstrated by Lord et al [82]. Furthermore, the correlation between performance on the 5R-STS test and the TUG test, as well as gait speed tests, adds further support for its validity, as highlighted by Schaubert and Bohannon [91].

Although the 5R-STS and 30CST tests involve identical movements, they are not interchangeable. The 5R-STS indicates lower limb speed and power for those with lower physical function post-TKA, while the 30CST measures lower limb endurance for those with higher physical function [92].

While outcome measures are often viewed as purely psychometric, their clinical relevance is established through their role as important drivers of long-term surgical success. For instance, a recent systematic review and meta-analysis by Sumbal et al [93] demonstrated that loss of muscle mass, or sarcopenia, is a significant risk factor for prosthetic loosening, which remains a primary indication for revision surgery. Furthermore, physical performance levels and quadriceps strength have been shown to be strongly associated with patient satisfaction and restoration of functional ability after TKA [94]. More recently, Akatsuka et al [95] reinforced this clinical relevance by reporting significant correlations between quadriceps muscle mass and postoperative satisfaction, suggesting that these objective measures are predictive of how a patient perceives their surgical result.

## Discussion

### Principal findings

A range of assessment tools is available to evaluate recovery following TKA, each with distinct advantages and limitations. This review highlights the use, strengths, and constraints of these measures and recommends a comprehensive, multidimensional evaluation framework. Such an approach enhances the accuracy and clinical relevance of recovery assessments by triangulating data across patient-reported outcomes, strength measures, functional performance, and muscle mass.

Among muscle mass assessment techniques, DXA remains the gold standard due to its high precision and reliability. However, alternative methods such as BIA offer a noninvasive, lower-cost option suitable for clinical settings, despite reduced accuracy and sensitivity to minor changes in muscle composition.

PROMs provide valuable insights into patient perspectives. The OKS is widely employed due to its ease of use, absence of ceiling or floor effects in the short term, and extensive validation in clinical research. KOOS, while broader in scope, often demonstrates ceiling effects and a higher completion burden. WOMAC overlaps conceptually but lacks OKS’s responsiveness in early recovery. KSS combines patient and clinician input but is less feasible for routine use due to scoring complexity and reliance on in-person assessment. OKS offers the optimal balance of validity, efficiency, and clinical use in TKA follow-up. Nevertheless, PROMs alone do not provide objective strength assessments, which are critical for a comprehensive evaluation.

In contrast, IKD offers precise quantification of muscle strength, yet its high cost and operational complexity restrict its widespread application. As a practical alternative, PFD provides a reliable, cost-effective, and portable means of assessing muscle strength, making it highly suitable for routine clinical practice.

Performance-based measures such as the 5R-STS test offer objective, time-efficient insights into lower limb function. Compared to the TUG, 6MWT, or SCT, the 5R-STS requires less space, is less influenced by cardiovascular limitations, and is more feasible for patients with early post-op mobility impairments. It also shows strong correlation with knee extension strength and mobility metrics, enhancing its predictive use in post-TKA recovery.

**Figure 2.**
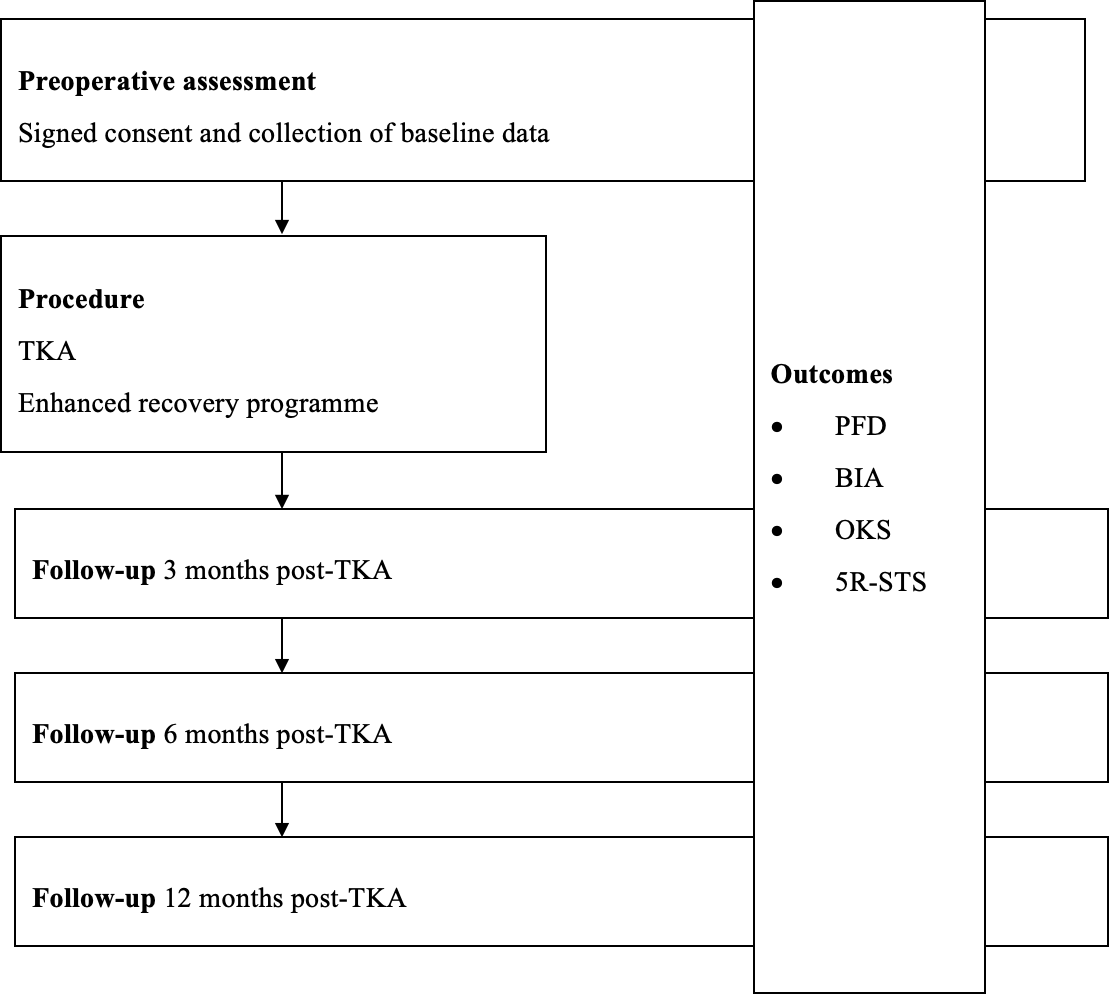
Clinical workflow algorithm for integrating recommended outcome measures throughout the total knee arthroplasty (TKA) pathway. The flowchart outlines the timing and selection of assessment tools from the preoperative assessment appointment to the final follow-up. 5R-STS: 5-Repetition Sit-to-Stand; BIA: bioelectrical impedance analysis; OKS: Oxford Knee Score; PFD: portable fixed dynamometer.

This specific combination, OKS, PFD, BIA, and 5R-STS is what we have recommended in post-TKA follow-up (Figure 2) because it balances psychometric validity, clinical feasibility, and comprehensive recovery profiling. It provides a more complete view of TKA recovery than any individual tool. While alternatives like the 6MWT or SCT yield valuable data, their requirements for time, space, or greater cardiopulmonary reserve make them less practical for routine follow-up or for patients with limited mobility or comorbidities. Likewise, the TUG, though simple, primarily reflects balance and walking ability but lacks sensitivity to muscle power or patient satisfaction domains.

### Limitations

Finally, it is important to note that the interpretability and use of these tools may vary across populations. For instance, sarcopenia, obesity, or cardiopulmonary conditions may skew performance-based results or BIA readings, requiring clinicians to contextualize outcomes based on age, BMI, and comorbid burden. By triangulating across these assessments, clinicians can reduce bias from any single measure and develop a more individualized recovery evaluation.

### Conclusions

Throughout this paper, evidence suggests that incorporating muscle-based and performance-based measures alongside PROMs is essential for a comprehensive and clinically relevant assessment of TKA recovery. However, future research should ensure there are multicenter trials to validate the integration of the proposed assessments, as well as consensus agreement with clinicians (ie, through a Delphi approach) which translates into guidelines. Additionally, a greater understanding of intrinsic skeletal muscle (mal)adaptations following TKA may provide valuable insight into the mechanisms underlying persistent functional limitations.

## Supplementary material

10.2196/84011 Multimedia Appendix 1 Comparative analysis of outcome measures used in total knee arthroplasty (TKA), including patient-reported outcomes, muscle strength, muscle mass, and physical performance tools. Comparison criteria include measurement type, reliability, validity, ease of use, clinical relevance, limitations, and best use case.

## Abbreviations

1-RM: 1–repetition maximum
30CST: 30-Second Chair Stand Test
5R-STS: 5-Repetition Sit-to-Stand
6MWT: 6-Minute Walk Test
ADL: activities of daily living
BIA: bioelectrical impedance analysis
COSMIN: Consensus-Based Standards for the Selection of Health Measurement Instruments
CT: computed tomography
DXA: dual-energy X-ray absorptiometry
ICC: intraclass correlation coefficient
KOOS: Knee Injury and Osteoarthritis Outcome Score
KSS: Knee Society Clinical Rating System
MRI: magnetic resonance imaging
OKS: Oxford Knee Score
PFD: portable fixed dynamometer
PROM: patient-related outcome measure
ROM: range of motion
SCT: Stair Climb Test
STS: Sit-to-Stand
TKA: total knee arthroplasty
TUG: Time Up and Go
WOMAC: Western Ontario and McMaster Universities Osteoarthritis

## References

[1] Neogi T (2013). The epidemiology and impact of pain in osteoarthritis. Osteoarthritis Cartilage.

[2] Vos T, Lim SS, Abbafati C (2020). Global burden of 369 diseases and injuries in 204 countries and territories, 1990-2019: a systematic analysis for the Global Burden of Disease Study 2019. Lancet.

[3] Jüni P, Reichenbach S, Dieppe P (2006). Osteoarthritis: Rational approach to treating the individual. Best Pract Res Clin Rheumatol.

[4] Rissolio L, Sabatini L, Risitano S (2021). Is it the surgeon, the patient, or the device? A comprehensive clinical and radiological evaluation of factors influencing patient satisfaction in 648 total knee arthroplasties. J Clin Med.

[5] Lewis GN, Rice DA, Rashid U, McNair PJ, Kluger MT, Somogyi AA (2023). Trajectories of pain and function outcomes up to 5 to 8 years following total knee arthroplasty. J Arthroplasty.

[6] Tungtrongjit Y, Weingkum P, Saunkool P (2012). The effect of preoperative quadriceps exercise on functional outcome after total knee arthroplasty. J Med Assoc Thai.

[7] Vajapey SP, Morris J, Spitzer AI, Glassman AH, Greco NJ, Li M (2020). Outcome reporting patterns in total knee arthroplasty: a systematic review. J Clin Orthop Trauma.

[8] Mizner RL, Petterson SC, Clements KE, Zeni JA, Irrgang JJ, Snyder-Mackler L (2011). Measuring functional improvement after total knee arthroplasty requires both performance-based and patient-report assessments: a longitudinal analysis of outcomes. J Arthroplasty.

[9] Naili JE, Iversen MD, Esbjörnsson AC (2017). Deficits in functional performance and gait one year after total knee arthroplasty despite improved self-reported function. Knee Surg Sports Traumatol Arthrosc.

[10] Dawson J, Fitzpatrick R, Murray D, Carr A (1998). Questionnaire on the perceptions of patients about total knee replacement. J Bone Joint Surg Br.

[11] Insall JN, Dorr LD, Scott RD, Scott WN (1989). Rationale of the Knee Society clinical rating system. Clin Orthop Relat Res.

[12] Bellamy N, Buchanan WW, Goldsmith CH, Campbell J, Stitt LW (1988). Validation study of WOMAC: a health status instrument for measuring clinically important patient relevant outcomes to antirheumatic drug therapy in patients with osteoarthritis of the hip or knee. J Rheumatol.

[13] Roos EM, Roos HP, Lohmander LS, Ekdahl C, Beynnon BD (1998). Knee Injury and Osteoarthritis Outcome Score (KOOS)—development of a self-administered outcome measure. J Orthop Sports Phys Ther.

[14] Martimbianco ALC, Calabrese FR, Iha LAN, Petrilli M, Lira Neto O, Carneiro Filho M (2012). Reliability of the “American Knee Society Score” (AKSS) [Article in Portuguese]. Acta Ortop Bras.

[15] Hodges PW, Smeets RJ (2015). Interaction between pain, movement, and physical activity: short-term benefits, long-term consequences, and targets for treatment. Clin J Pain.

[16] Merkle SL, Sluka KA, Frey-Law LA (2020). The interaction between pain and movement. J Hand Ther.

[17] Ghanem E, Pawasarat I, Lindsay A (2010). Limitations of the Knee Society Score in evaluating outcomes following revision total knee arthroplasty. J Bone Joint Surg Am.

[18] Vogel N, Kaelin R, Rychen T, Wendelspiess S, Müller-Gerbl M, Arnold MP (2024). Comparison of responsiveness of patient-reported outcome measures after total knee arthroplasty. J Arthroplasty.

[19] Bach CM, Nogler M, Steingruber IE (2002). Scoring systems in total knee arthroplasty. Clin Orthop Relat Res.

[20] Karimijashni M, Abtahi F, Abbasalipour S (2024). Functional patient-reported outcome measures after hip or knee arthroplasty: a systematic review and content analysis using the International Classification of Functioning, Disability, and Health. Arthritis Care Res (Hoboken).

[21] Noble PC, Scuderi GR, Brekke AC (2012). Development of a new Knee Society scoring system. Clin Orthop Relat Res.

[22] Scuderi GR, Bourne RB, Noble PC, Benjamin JB, Lonner JH, Scott WN (2012). The new Knee Society knee scoring system. Clin Orthop Relat Res.

[23] Siljander MP, McQuivey KS, Fahs AM, Galasso LA, Serdahely KJ, Karadsheh MS (2018). Current trends in patient-reported outcome measures in total joint arthroplasty: a study of 4 major orthopaedic journals. J Arthroplasty.

[24] Ritter MA, Davis KE, Davis P (2013). Preoperative malalignment increases risk of failure after total knee arthroplasty. J Bone Joint Surg Am.

[25] Capin JJ, Bade MJ, Jennings JM, Snyder-Mackler L, Stevens-Lapsley JE (2022). Total knee arthroplasty assessments should include strength and performance-based functional tests to complement range-of-motion and patient-reported outcome measures. Phys Ther.

[26] Ben-Shlomo Y, Blom A, Boulton C (2022). The National Joint Registry 19th annual report 2022. https://www.njrcentre.org.uk/wp-content/uploads/NJR-Supported-Research.pdf.

[27] Lee MK, Naessens JM, Eton DT, O’Byrne TJ, Nyman MA (2021). Functional outcomes and health-related quality of life before and after primary total knee replacement for patients from diverse geographic regions. J Arthroplasty.

[28] Beard DJ, Knezevic K, Al-Ali S, Dawson J, Price AJ (2010). The use of outcome measures relating to the knee. Orthop Trauma.

[29] Harris K, Lim CR, Dawson J, Fitzpatrick R, Beard DJ, Price AJ (2017). The Oxford Knee Score and its subscales do not exhibit a ceiling or a floor effect in knee arthroplasty patients: an analysis of the National Health Service PROMs data set. Knee Surg Sports Traumatol Arthrosc.

[30] Marx RG, Jones EC, Atwan NC, Closkey RF, Salvati EA, Sculco TP (2005). Measuring improvement following total hip and knee arthroplasty using patient-based measures of outcome. J Bone Joint Surg Am.

[31] Beard DJ, Davies LJ, Cook JA (2019). The clinical and cost-effectiveness of total versus partial knee replacement in patients with medial compartment osteoarthritis (TOPKAT): 5-year outcomes of a randomised controlled trial. Lancet.

[32] Escobar A, Gonzalez M, Quintana JM (2012). Patient acceptable symptom state and OMERACT-OARSI set of responder criteria in joint replacement. Identification of cut-off values. Osteoarthritis Cartilage.

[33] Berth A, Urbach D, Awiszus F (2002). Improvement of voluntary quadriceps muscle activation after total knee arthroplasty. Arch Phys Med Rehabil.

[34] Bellamy N (2002). WOMAC: A 20-year experiential review of a patient-centered self-reported health status questionnaire. J Rheumatol.

[35] Roos EM, Toksvig-Larsen S (2003). Knee injury and Osteoarthritis Outcome Score (KOOS)—validation and comparison to the WOMAC in total knee replacement. Health Qual Life Outcomes.

[36] Antosh IJ, Svoboda SJ, Peck KY, Garcia EJ, Cameron KL (2018). Change in KOOS and WOMAC scores in a young athletic population with and without anterior cruciate ligament injury. Am J Sports Med.

[37] Collins NJ, Prinsen CAC, Christensen R, Bartels EM, Terwee CB, Roos EM (2016). Knee injury and Osteoarthritis Outcome Score (KOOS): systematic review and meta-analysis of measurement properties. Osteoarthritis Cartilage.

[38] Wang Y, Yin M, Zhu S, Chen X, Zhou H, Qian W (2021). Patient-reported outcome measures used in patients undergoing total knee arthroplasty. Bone Joint Res.

[39] Harris K, Dawson J, Gibbons E (2016). Systematic review of measurement properties of patient-reported outcome measures used in patients undergoing hip and knee arthroplasty. Patient Relat Outcome Meas.

[40] Alviar MJ, Olver J, Brand C (2011). Do patient-reported outcome measures in hip and knee arthroplasty rehabilitation have robust measurement attributes? A systematic review. J Rehabil Med.

[41] Collins NJ, Roos EM (2012). Patient-reported outcomes for total hip and knee arthroplasty: commonly used instruments and attributes of a “good” measure. Clin Geriatr Med.

[42] Gerrits KH, Beltman MJ, Koppe PA (2009). Isometric muscle function of knee extensors and the relation with functional performance in patients with stroke. Arch Phys Med Rehabil.

[43] Maffiuletti NA (2010). Assessment of hip and knee muscle function in orthopaedic practice and research. J Bone Joint Surg Am.

[44] Hyun CW, Han EY, Im SH (2015). Hemiparetic knee extensor strength and balance function are predictors of ambulatory function in subacute stroke patients. Ann Rehabil Med.

[45] Norris R, Morrison S, Price A (2024). Inline dynamometry provides reliable measurements of quadriceps strength in healthy and ACL-reconstructed individuals and is a valid substitute for isometric electromechanical dynamometry following ACL reconstruction. Knee.

[46] Lienhard K, Lauermann SP, Schneider D, Item-Glatthorn JF, Casartelli NC, Maffiuletti NA (2013). Validity and reliability of isometric, isokinetic and isoinertial modalities for the assessment of quadriceps muscle strength in patients with total knee arthroplasty. J Electromyogr Kinesiol.

[47] Ieiri A, Tushima E, Ishida K, Inoue M, Kanno T, Masuda T (2015). Reliability of measurements of hip abduction strength obtained with a hand-held dynamometer. Physiother Theory Pract.

[48] Bohannon RW, Kindig J, Sabo G, Duni AE, Cram P (2012). Isometric knee extension force measured using a handheld dynamometer with and without belt-stabilization. Physiother Theory Pract.

[49] Bohannon RW, Pritchard RO, Glenney SS (2013). Portable belt–stabilized hand-held dynamometry set-up for measuring knee extension force. Isokinet Exerc Sci.

[50] Martins J, da Silva JR, da Silva MRB, Bevilaqua-Grossi D (2017). Reliability and validity of the belt-stabilized handheld dynamometer in hip- and knee-strength Tests. J Athl Train.

[51] Toonstra J, Mattacola CG (2013). Test-retest reliability and validity of isometric knee-flexion and -extension measurement using 3 methods of assessing muscle strength. J Sport Rehabil.

[52] Abdalla PP, Carvalho ADS, Dos Santos AP (2020). One-repetition submaximal protocol to measure knee extensor muscle strength among older adults with and without sarcopenia: a validation study. BMC Sports Sci Med Rehabil.

[53] Horstmann H, Medico P, Lasch F, Krutsch W, Weber-Spickschen TS (2020). Simplified measurement of maximum strength after knee surgery: application-based knee-training device compared to isokinetic testing. Open Access J Sports Med.

[54] Whiteley R, Jacobsen P, Prior S, Skazalski C, Otten R, Johnson A (2012). Correlation of isokinetic and novel hand-held dynamometry measures of knee flexion and extension strength testing. J Sci Med Sport.

[55] Lauermann SP, Lienhard K, Item-Glatthorn JF, Casartelli NC, Maffiuletti NA (2014). Assessment of quadriceps muscle weakness in patients after total knee arthroplasty and total hip arthroplasty: methodological issues. J Electromyogr Kinesiol.

[56] Tsukasaki K, Matsui Y, Arai H (2020). Association of muscle strength and gait speed with cross-sectional muscle area determined by mid-thigh computed tomography—a comparison with skeletal muscle mass measured by dual-energy X-ray absorptiometry. J Frailty Aging.

[57] Riviati N, Indra B (2023). Relationship between muscle mass and muscle strength with physical performance in older adults: a systematic review. SAGE Open Med.

[58] Heymsfield SB, Adamek M, Gonzalez MC, Jia G, Thomas DM (2014). Assessing skeletal muscle mass: historical overview and state of the art. J Cachexia Sarcopenia Muscle.

[59] Binay Safer V, Safer U (2013). Usefulness and limitations of single-slice computed tomography analysis at the third lumbar region in the assessment of sarcopenia. Crit Care.

[60] Gibson DJ, Burden ST, Strauss BJ, Todd C, Lal S (2015). The role of computed tomography in evaluating body composition and the influence of reduced muscle mass on clinical outcome in abdominal malignancy: a systematic review. Eur J Clin Nutr.

[61] Lemos T, Gallagher D (2017). Current body composition measurement techniques. Curr Opin Endocrinol Diabetes Obes.

[62] Strandberg S, Wretling ML, Wredmark T, Shalabi A (2010). Reliability of computed tomography measurements in assessment of thigh muscle cross-sectional area and attenuation. BMC Med Imaging.

[63] Erlandson MC, Lorbergs AL, Mathur S, Cheung AM (2016). Muscle analysis using pQCT, DXA and MRI. Eur J Radiol.

[64] Heymsfield SB, Gonzalez MC, Lu J, Jia G, Zheng J (2015). Skeletal muscle mass and quality: evolution of modern measurement concepts in the context of sarcopenia. Proc Nutr Soc.

[65] Prado CMM, Heymsfield SB (2014). Lean tissue imaging: a new era for nutritional assessment and intervention. JPEN J Parenter Enteral Nutr.

[66] Lustgarten MS, Fielding RA (2011). Assessment of analytical methods used to measure changes in body composition in the elderly and recommendations for their use in phase II clinical trials. J Nutr Health Aging.

[67] Maden-Wilkinson TM, Degens H, Jones DA, McPhee JS (2013). Comparison of MRI and DXA to measure muscle size and age-related atrophy in thigh muscles. J Musculoskelet Neuronal Interact.

[68] Kim J, Wang Z, Heymsfield SB, Baumgartner RN, Gallagher D (2002). Total-body skeletal muscle mass: estimation by a new dual-energy X-ray absorptiometry method. Am J Clin Nutr.

[69] Taylor AE, Kuper H, Varma RD (2012). Validation of dual energy X-ray absorptiometry measures of abdominal fat by comparison with magnetic resonance imaging in an Indian population. PLoS One.

[70] Hangartner TN, Warner S, Braillon P, Jankowski L, Shepherd J (2013). The official positions of the International Society for Clinical Densitometry: acquisition of dual-energy X-ray absorptiometry body composition and considerations regarding analysis and repeatability of measures. J Clin Densitom.

[71] Toombs RJ, Ducher G, Shepherd JA, De Souza MJ (2012). The impact of recent technological advances on the trueness and precision of DXA to assess body composition. Obesity (Silver Spring).

[72] Johnson Stoklossa CA, Forhan M, Padwal RS, Gonzalez MC, Prado CM (2016). Practical considerations for body composition assessment of adults with class ii/iii obesity using bioelectrical impedance analysis or dual-energy x-ray absorptiometry. Curr Obes Rep.

[73] Khalil SF, Mohktar MS, Ibrahim F (2014). The theory and fundamentals of bioimpedance analysis in clinical status monitoring and diagnosis of diseases. Sensors (Basel).

[74] Marra M, Sammarco R, De Lorenzo A (2019). Assessment of body composition in health and disease using bioelectrical impedance analysis (BIA) and dual energy x-ray absorptiometry (DXA): a critical overview. Contrast Media Mol Imaging.

[75] Buckinx F, Reginster JY, Dardenne N (2015). Concordance between muscle mass assessed by bioelectrical impedance analysis and by dual energy x-ray absorptiometry: a cross-sectional study. BMC Musculoskelet Disord.

[76] Ling CHY, de Craen AJM, Slagboom PE (2011). Accuracy of direct segmental multi-frequency bioimpedance analysis in the assessment of total body and segmental body composition in middle-aged adult population. Clin Nutr.

[77] Jette DU, Hunter SJ, Burkett L (2020). Physical therapist management of total knee arthroplasty. Phys Ther.

[78] Dobson F, Hinman RS, Roos EM (2013). OARSI recommended performance-based tests to assess physical function in people diagnosed with hip or knee osteoarthritis. Osteoarthritis Cartilage.

[79] (2017). Rehabilitative care best practices for patients with primary hip & knee replacements. https://rehabcarealliance.ca/wp-content/uploads/2025/03/TJR_Framework.pdf.

[80] Studenski S, Perera S, Patel K (2011). Gait speed and survival in older adults. JAMA.

[81] Fenner V, Behrend H, Kuster MS (2014). Whole body gait function during stair ascending and level walking in patients following total knee arthroplasty. Int J Phys Med Rehabil.

[82] Lord SR, Murray SM, Chapman K, Munro B, Tiedemann A (2002). Sit-to-stand performance depends on sensation, speed, balance, and psychological status in addition to strength in older people. J Gerontol A Biol Sci Med Sci.

[83] Mizner RL, Petterson SC, Snyder-Mackler L (2005). Quadriceps strength and the time course of functional recovery after total knee arthroplasty. J Orthop Sports Phys Ther.

[84] Bohannon RW (2011). Test-retest reliability of the five-repetition sit-to-stand test: a systematic review of the literature involving adults. J Strength Cond Res.

[85] Dobson F, Hinman RS, Hall M (2017). Reliability and measurement error of the Osteoarthritis Research Society International (OARSI) recommended performance-based tests of physical function in people with hip and knee osteoarthritis. Osteoarthritis Cartilage.

[86] Hoens A, Westby M, Longstaff S, Duggan M (2016). Total Joint Arthroplasty and Outcome Measures (TJAOM) toolkit. The University of British Columbia.

[87] Zeni JA, Axe MJ, Snyder-Mackler L (2010). Clinical predictors of elective total joint replacement in persons with end-stage knee osteoarthritis. BMC Musculoskelet Disord.

[88] Bergquist R, Weber M, Schwenk M (2019). Performance-based clinical tests of balance and muscle strength used in young seniors: a systematic literature review. BMC Geriatr.

[89] Gill S, Hely R, Page RS, Hely A, Harrison B, Landers S (2022). Thirty second chair stand test: test-retest reliability, agreement and minimum detectable change in people with early-stage knee osteoarthritis. Physiother Res Int.

[90] Klukowska AM, Staartjes VE, Vandertop WP, Schröder ML (2021). Five-repetition sit-to-stand test performance in healthy individuals: reference values and predictors from 2 prospective cohorts. Neurospine.

[91] Schaubert KL, Bohannon RW (2005). Reliability and validity of three strength measures obtained from community-dwelling elderly persons. J Strength Cond Res.

[92] McCarthy EK, Horvat MA, Holtsberg PA, Wisenbaker JM (2004). Repeated chair stands as a measure of lower limb strength in sexagenarian women. J Gerontol A Biol Sci Med Sci.

[93] Sumbal R, Abbas M, Sheikh SM, Sumbal A (2024). Prevalence and clinical impact of sarcopenia in patients undergoing total joint arthroplasty: a systematic review and a meta-analysis. J Arthroplasty.

[94] Furu M, Ito H, Nishikawa T (2016). Quadriceps strength affects patient satisfaction after total knee arthroplasty. J Orthop Sci.

[95] Akatsuka Y, Teramoto A, Takashima H, Okada Y, Watanabe K, Yamashita T (2023). Relationships of cross-sectional area of the thigh muscles before or after total knee arthroplasty with postoperative pain or patient satisfaction: a retrospective, exploratory study. Asia Pac J Sports Med Arthrosc Rehabil Technol.

