# Supplementary material for: Multidimensional Assessment of Recovery After Total Knee Arthroplasty in Clinical Practice: Critical Narrative Review
Source: JMIR Perioper Med. 2026 Feb 25;9:e84011. doi: 10.2196/84011 (PMC12935420; doi:10.2196/84011)
Supplement: Multimedia Appendix 1 [file periop-v9-e84011-s001.docx]

| **Comparison** | **PROMs (KSS, WOMAC, KOOS, OKS)** | **Muscle Strength Tests (IKD, PFD, HHD)** | **Muscle Mass Tests (CT, MRI, DXA, BIA)** | **Physical Performance Tests (TUG, 6MWT, 5R-STS, SCT)** |
| --- | --- | --- | --- | --- |
| **Measurement Type** | Subjective (patient-reported pain, function) | Objective (muscle force output) | Objective (muscle cross-sectional area or mass) | Objective (functional movement performance) |
| **Reliability** | High test-retest reliability but can be influenced by patient perception | High reliability but dependent on test consistency and patient effort | High for CT, MRI, and DXA; moderate for BIA (affected by hydration) | High for TUG, 6MWT, 5R-STS |
| **Validity** | Valid for subjective function but may not correlate with actual muscle strength or mobility | IKD, highest validity. PFD, good validity with proper fixation. HHD, lower validity | Highly valid for assessing atrophy or hypertrophy | Strong correlation with mobility and functional independence |
| **Ease of Use** | Simple, non-invasive, patient-friendly | Requires equipment; portable dynamometers are easier than isokinetic ones | CT/MRI are expensive; DXA is more accessible; BIA is easiest | Quick, requires minimal equipment (e.g., chair, stopwatch) |
| **Clinical Relevance** | Useful for tracking patient-perceived recovery | Directly assesses quadriceps and hamstring strength, critical for TKA recovery | Helps detect muscle loss post-TKA, which can affect long-term function | Strong predictor of fall risk, mobility, and independence |
| **Limitations** | Subjective, may not reflect real functional capacity | Requires patient cooperation; costly for isokinetic dynamometers | Expensive (CT, MRI), radiation exposure (CT, DXA), less precise (BIA) | Can be influenced by patient motivation, fatigue, or comorbidities |
| **Best Use Case** | Tracking patient-perceived progress | Measuring post-TKA quadriceps/hamstring recovery | Evaluating long-term muscle loss and sarcopenia | Assessing mobility and real-world function |

***Table 1***: Comparative analysis of outcome measures used in TKA, including patient-reported outcomes, muscle strength, muscle mass, and physical performance tools. Comparison criteria include measurement type, reliability, validity, ease of use, clinical relevance, limitations, and best use case.
